# Empirical ratio of the combined use of S-ketamine and propofol in electroconvulsive therapy and its impact on seizure quality

**DOI:** 10.1007/s00406-020-01170-7

**Published:** 2020-07-22

**Authors:** Alexander Sartorius, Juliane Beuschlein, Dmitry Remennik, Anna-Maria Pfeifer, Sebastian Karl, Jan Malte Bumb, Suna Su Aksay, Laura Kranaster, Christoph Janke

**Affiliations:** 1 Department of Psychiatry and Psychotherapy, Medical Faculty Mannheim, Central Institute of Mental Health, Heidelberg University, J5, 68159 Mannheim, Germany; 2 Department of Anaesthesiology and Surgical Intensive Care Medicine, Medical Faculty Mannheim, Heidelberg University, Mannheim, Germany; 3 Department of Addictive Behaviour and Addiction Medicine, Medical Faculty Mannheim, Central Institute of Mental Health, Heidelberg University, Mannheim, Germany

**Keywords:** ECT, Electroconvulsive therapy, Ketofol, (S-) ketamine, Propofol, Seizure quality, Recovery time

## Abstract

Electroconvulsive therapy (ECT) is an effective treatment for depressive disorders. In certain cases, ECT-associated anaesthesia can be improved by the use of ketofol (i.e., S-ketamine + propofol). We aimed to evaluate the empirical mixing ratio of ketofol in these cases for better clinical implementation. We retrospectively investigated *n* = 52 patients who received 919 ECT sessions with S-ketamine plus propofol as anaesthetic agents. Several anaesthesia and ECT-related parameters including doses of S-ketamine and propofol were analysed. The mean empirically determined S-ketamine/propofol ratio was 1.38 (SD ± 0.57) for 919 individual ECT sessions and 1.52 (SD ± 0.62) for 52 patients, respectively. The mean relative dose was 0.72 (± 0.18) mg/kg S-ketamine and 0.54 (± 0.21) mg/kg propofol. Higher propofol dose was associated with poorer seizure quality. Seizure quality and time in recovery room were significantly influenced by age. Ketofol could be an option to exploit the advantageous qualities of S-ketamine and propofol, if both doses are reduced compared with single use of S-ketamine or propofol. Patients with poor seizure quality may benefit from lower propofol doses, which are applicable by the addition of ketamine. An empirically determined mixing ratio in favour of ketamine turned out to be preferable in a clinical setting. Recovery time was primarily prolonged by higher age rather than by ketamine dose, which had previously often been associated with a prolonged monitoring time in the recovery room. These new findings could improve electroconvulsive therapy and should be replicated in a prospective manner.

## Introduction

In 2015, depressive disorders affected 322 million people worldwide, representing 4.4% of the world’s population [1]. Electroconvulsive therapy (ECT) is presumably the most effective treatment for depression [2]. Especially in case of suicidality and other threatening conditions like refusal to eat and drink or grave psychomotor retardation, ECT is the treatment of choice [3].

Anaesthesia is an indispensable part of ECT treatment to avoid awareness of muscle relaxation, which in turn is needed to prevent injuries caused by motor seizure [4]. The main hypnotic agents applied in ECT anaesthesia are barbiturates, etomidate, propofol and ketamine. Different characteristics of each agent can influence ECT effectivity and/or tolerability [5]. Apart from ketamine and etomidate, all aforementioned agents possess anticonvulsive properties [5, 6]. In numerous studies propofol yielded the shortest seizure durations [7] and inferior effectiveness [8], yet it was also associated with a better cardiovascular tolerability [9].

Propofol activates GABA-associated channels and central inhibition leads to anticonvulsive features [10]. These anticonvulsive properties might affect seizure quality in a negative way. Ketamine in contrast is typically not anticonvulsive and has a preferable influence on seizure quality [6]. Besides shorter seizure duration and inferior effectiveness of ECT [8], higher rates of failure in seizure induction, need of restimulation and bilateral stimulation have been reported with propofol compared to methohexital [11]. Therefore, it was suggested to not use more than 1 mg propofol per kg bodyweight not to shorten seizure duration [12]. Propofol’s anticonvulsive effects often make a higher stimulation dose necessary to achieve sufficient seizure quality [13, 14], which may lead to more cognitive side effects [13]. Recent studies suggested to prolong the time interval between hypnotic induction and ECT stimulation to overcome the initial action of propofol as an anticonvulsant [15, 16], which has also been suggested for other anticonvulsants like thiopental [17].

S-ketamine is an N-methyl-d-aspartate (NMDA) receptor antagonist and it does not negatively influence seizure quality because it has no anticonvulsive effects [6] at least for the dose ranges used within this study. Especially patients with non-sufficient seizure quality with other hypnotic agents might benefit from the use of ketamine [18]. Moreover, it might have synergistic antidepressive effects [19] or even improve cognitive outcome [19, 20]. Compared to propofol, ketamine might show an earlier improvement of depressive symptoms, without improving remission rate [21].

The idea of ketofol as a mixture of S-ketamine and propofol is to combine the advantages of both hypnotic agents. Prospective studies investigating ketofol in ECT delivered promising results regarding tolerability of the procedure and seizure quality (e.g., [22]. However, investigations to find the best mixing ratio of ketofol have not been conducted to date. A commercially available 1:1 mixture might not reflect the optimum for each individual patient. This paper aims to narrow this gap in research by empirically evaluating a more favourable mixing ratio of ketofol to optimize future anaesthesia in ECT.

## Materials and methods

Retrospectively, 919 ECT sessions performed in the Department of Psychiatry and Psychotherapy at the Central Institute of Mental Health, Mannheim, Germany between 2016 and 2018 were analysed. Approximately 1/3 of all patients treated with ECT within the inclusion period received a mixture of propofol and S-ketamine. S-ketamine in patients not treated with additional propofol (and not included in this study) is typically given at doses between 0.9 and 1.0 mg/kg. The only inclusion criterion was the use of a mixture of propofol and S-ketamine as hypnotic agents. The Thymatron IV device (Somatics, LLC. Lake Bluff, IL, USA) was used performing ECT sessions. Pulse width was chosen according to the “double dose” program setting of the Thymatron IV device.

Anaesthetics and their doses were chosen according to the anaesthesiologist’s personal clinical experience and adjusted within ECT period if necessary. For this reason and due to our pre-existing and published experience [6, 23] we used only S-Ketamine and not the racemic mixture (ketamine). Initial anaesthesia was induced with S-ketamine alone with typically 1 mg/kg body weight in most cases. However, some patients started with a combination of propofol and S-ketamine (ketofol), other received additional propofol during the ECT series for different reasons like post-ictal agitation, psychomimetic side effects in the recovery room, high blood pressure peaks or anaesthesia related anxiety. Propofol was typically added with doses below 0.5 mg/kg while S-ketamine dose was reduced.

Based on our experience with monitoring of the depth of anaesthesia [24] and based on literature suggesting at least 2.5 min [17, 25] we used a 4 min time interval between anaesthesia induction and ECT stimulation as a routine interval in all patients. Propofol was injected before S-ketamine in all cases.

Only the treatments with combination of both anaesthetics were included in our study. The following parameters have been documented for each ECT session: S-ketamine and propofol dose (mg), electrode placement (unilateral or bilateral), stimulation dose (%), postictal suppression index (PSI) (%), midictal amplitude (μV), peak heart rate (beats/min), seizure duration (sec) based on EEG and EMG recordings and maximum sustained coherence (%) (coherence is defined by the correlation of ictal activity between both hemispheres) of the seizure.

After every ECT session patients were transferred to the recovery room, where postictal monitoring of each patient was conducted by a single trained ECT nurse. Patients left the recovery room when cardiorespiratory functions and orientation status returned to pre-ECT state. Orientation (time, place, situation, person) was routinely documented every 5 min. Total duration of stay in the recovery room (min) was documented.

### Seizure quality index (SQI)

To determine seizure quality we used two versions of seizure quality index (SQI) including concordance (the ratio between duration of motor response and EEG seizure duration), midictal amplitude, peak heart rate, maximum interhemispheric coherence, postictal suppression index (PSI) and seizure duration (EMG). Based on Hoyer et al. we classified an index regardless of the patient’s age [23]. In short, SQI was based on five conditions, which were concordance > 0.8, PSI > 0.8, maximal interhemispheric coherence > 0.9, peak heart rate > 125 bpm and midictal amplitude > 150 µV. Each fulfilled condition yielded one point, resulting in an SQI ranging from zero to five. The SQI_*K*_ by Kranaster et al. is similar, but limited to non-geriatric patients under 65 years [26]. Missing data for both indices were imputed using last observation carried on forward.

### Statistical analysis

All statistics were performed using StataSE (StataCorp, Texas 77,845, USA, version 15) at a significance level of *p* = 0.05. ECT sessions were referred to the respective patient. Multiple regression analyses and linear regression were performed to evaluate the influence of the mixing ratio of S-ketamine and propofol. STATA “collapse” converted the dataset into a dataset of means, e.g., if a patient received ten ECTs his/her dataset was reduced to a single mean value for each variable (like charge, anaesthesia dose, reorientation time, etc.). Potential covariates for time spent in recovery room were analysed by ANCOVA.

## Results

In total, 919 ECT sessions of 52 patients [29 men (55.8%) and 23 women (44.2%)] with an average age of 55.2 years have been analysed. The age range contained patients from 13 to 87 years (55.2 years ± SD 19.6). 2/3 of all patients were treated due to a major depressive episode, and 1/3 due to schizophrenia or catatonia. Mean stimulation dose was 534 mC. 446 ECT sessions (48.53%) were performed with unilateral and 473 (51.47%) with bilateral electrode placement. 4 (of 52) patients received maintenance ECT totaling in 172 (of 919) treatments. Detailed information and ictal parameters are shown in (Table 1) (collapsed data).

**Table 1:** Anaesthetic, ictal and postictal parameters

	n	Mean (± SD)	Min	Max
Anaesthetic parameters				
Absolute S-ketamine dose (mg)	52	57 (± 17)	30	89
Relative S-ketamine dose (mg/kg)	52	0.72 (± 0.18)	0.38	1.14
Absolute propofol dose(mg)	52	43 (± 18)	10	103
Relative propofol dose (mg/kg)	52	0.54 (± 0.21)	0.18	1.14
Ratio S-ketamine/propofol	52	1.52 (± 0.62)	0.75	4
Seizure parameters				
Stimulation dose (mC)	52	442 (± 233)	90	1008
PSI (%)	49	79 (± 15)	38	96
Midictal amplitude (μV)	52	163 (± 63)	43	279
Total coherence (%)	52	90 (± 8)	65	98
Peak heart rate (/min)	52	127 (± 20)	77	166
Seizure in EMG (sec)	52	26 (± 10)	9	61
Seizure in EEG (sec)	52	42 (± 14)	16	89
Concordance	52	0.63 (± 0.14)	0.29	0.97
SQI	49	2.52 (± 1.28)	0	4.91
SQI_K_	35	1.46 (± 0.81)	0.20	3
Recovery room				
Time in recovery room (min)	52	33.3 (± 8.0)	17.22	56.50

### Hypnotic agents and ketofol ratio

Using data from all 919 ECT sessions resulted in a mean empirical S-ketamine/propofol ratio of 1.38 (SD ± 0.57), whereas a ratio of 1.52 (SD ± 0.62) was calculated using collapsed data (*n* = 52). For the final ECT session S-ketamine/propofol ratio was 1.57 (± 0.68). The mean doses were 0.72 (± 0.18) mg/kg for S-ketamine and 0.54 (± 0.21) mg/kg for propofol using collapsed data.

30 out of 52 patients received urapidil (a standard drug to treat the post-ictal rise of blood pressure). Urapidil dosage showed a significant positive correlation with S-ketamine dose (mg/kg) (*p* = 0.024) in a linear least-squares regression.

### Influence on seizure quality

Regarding SQI, higher dose of propofol (mg/kg) correlated with lower seizure quality (SQI: *z* = − 4.48, *p* = 0.000, 95% CI: − 3.31–1.29; SQI_*K*_: *z* = − 3.07, *p* = 0.002, 95% CI: − 2.49–0.55). In contrast to this, ketamine dose did not have a significant impact on seizure quality (SQI: *p* = 0.121; SQI_*K*_: *p* = 0.073).

**Fig. 1:**
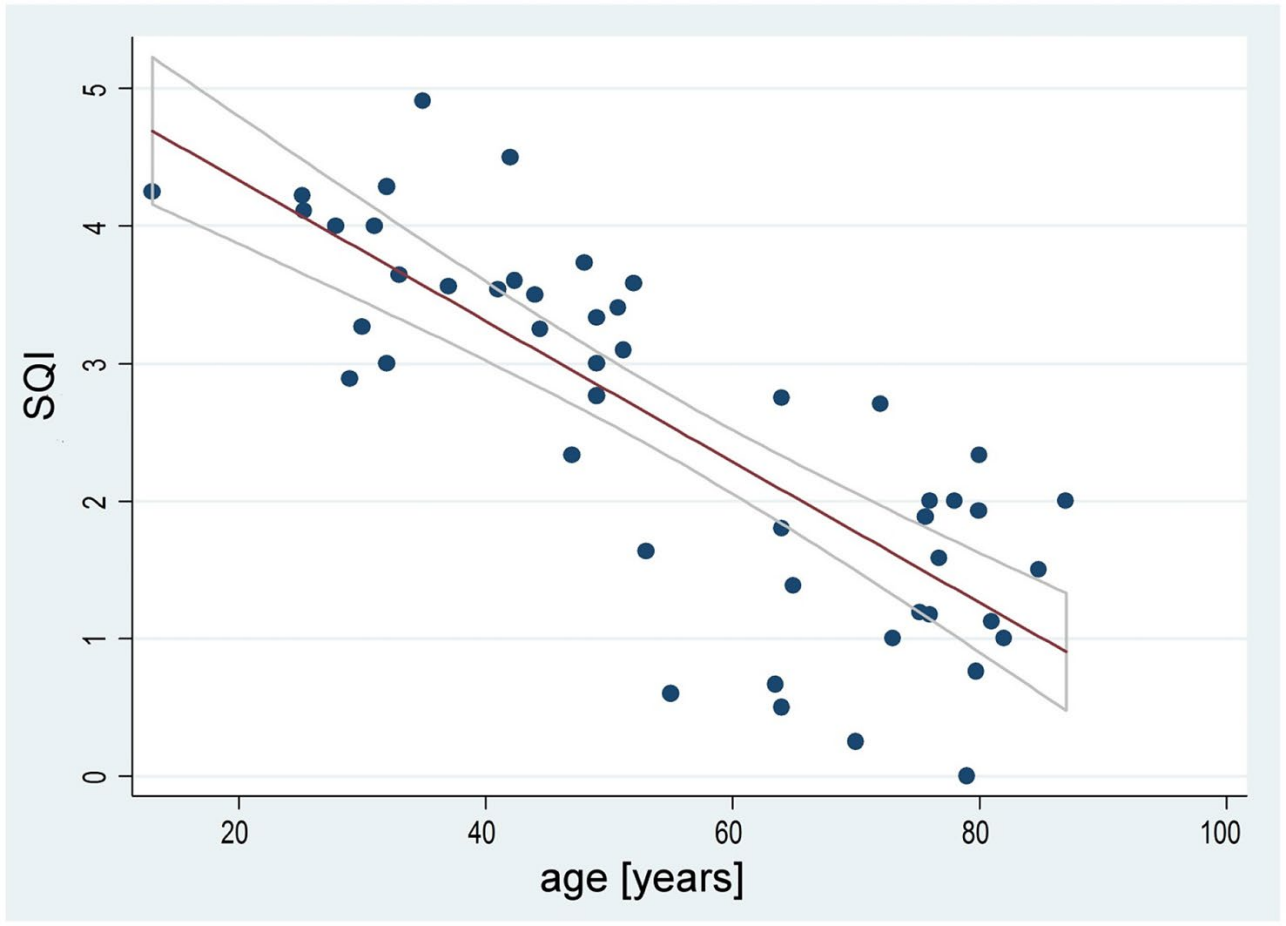
Negative correlation between age and SQI. Line fits represent linear regression and 95% confidence interval

Higher age had a negative influence on the patient’s SQI (*t* = − 27.66, *p* = 0.000, R-squared = 0.47, 95% CI: − 0.058–0.047), see (Fig. 1).

### Different SQI versions in comparison

Comparison of SQI_K_ and SQI for patients under the age of 65 years using linear least-squares regression analysis revealed a highly significant correlation (*t* = 15.57, *p* = 0.000, R-squared = 0.304, 95% CI: 0.54–0.69).

### Time in recovery room (min)

**Fig. 2:**
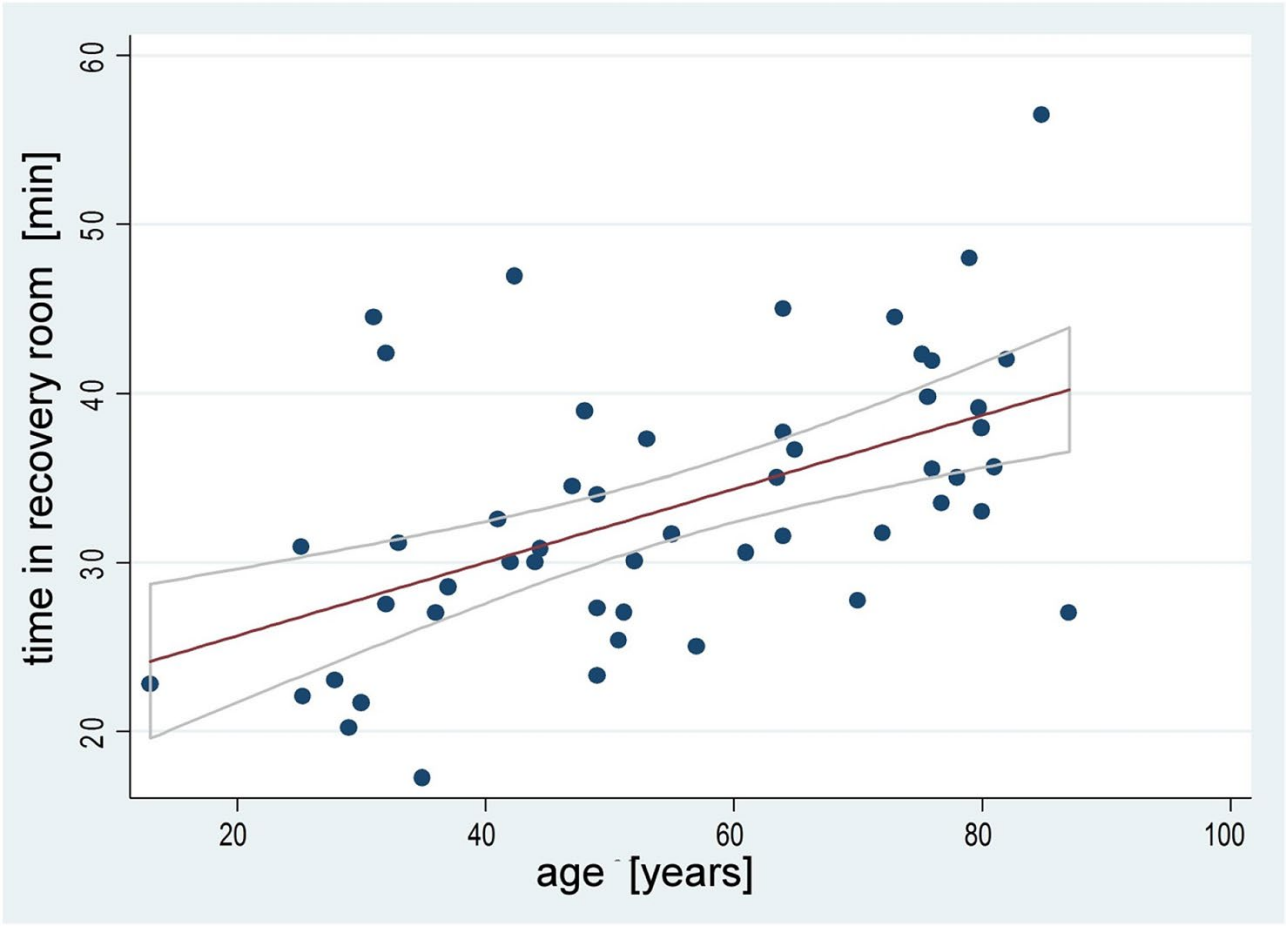
Positive correlation between age and time spent in recovery room. Line fits represent linear regression and 95% confidence interval

Time in recovery room was mainly influenced by the patient’s age. The conducted ANCOVA revealed higher age (*F* = 8.60, *p* = 0.0053) being an important covariate regarding prolonged time in recovery room. Other potential covariates, for example relative ketamine dose (mg/kg) (*F* = 0.00, *p* = 0.9517), did not show a significant correlation. The (post hoc) positive correlation between the patients’ age and time in recovery room is shown in (Fig. 2).

## Discussion

We retrospectively investigated empirical S-ketamine/propofol doses and ratio, and their relation to typical ECT parameters. S-ketamine/propofol doses and their adaptations during the ECT course were empirically driven by the idea of minimizing side effects (e.g., by reducing S-ketamine) and optimizing ECT (basically by reducing propofol due to its anticonvulsive potency). This clinical optimisation process led to different ratios and doses in individual patients.

A typical mixture of ketamine and propofol uses a 1:1 ratio [27], but there are also studies using ratios with an even higher proportion of propofol (e.g., 1:3) [28]. Regarding ECT it was recently suggested to use a ratio of ketamine and propofol of 1:1 [5]. This would correspond to a ratio of S-ketamine: propofol of 0.5:1 or 1:2 assuming that only S-ketamine is effective within the racemate. Our analysis showed a much higher mean empirically determined S-ketamine: propofol ratio of 1.38 (SD ± 0.57) for 919 individual ECT sessions or 1.52 (SD ± 0.62) for 52 patients (collapsed data). For the racemic mixture this would translate into a ketamine: propofol ratio of 2.76 and 3.04 (collapsed data), respectively.

Wang et al. compared ketamine (0.8 mg/kg), propofol (1.5 mg/kg) and ketofol (0.8 mg/kg) plus (1.5 mg/kg), respectively in a randomized trial and found ketamine and ketofol both to be associated with faster antidepressive effects, with ketamine alone being associated with more side effects (i.e., hypertension and fear) [29].

Yalcin et al. administered ketofol (1:1), propofol and ketamine, but with a roughly 50% reduction of both substances in the ketofol group [22]. Motor seizure time was significantly reduced in the propofol group while parameters of recovery were best in the propofol and ketofol groups.

In our study, propofol dose showed a negative influence on seizure quality for both indices (SQI and SQI_*K*_). This finding emphasizes propofol’s anticonvulsive properties even at lower doses (i.e., 0.54 mg/kg). Despite the definition of seizure quality between SQI and SQI_K_ this finding was stable between both definitions.

Therefore, a mixing ratio in favour of (S-) ketamine could be more advantageous.

Age is an important covariate concerning seizure quality. Higher age had a negative influence on the patient’s SQI. This finding has been reported by many studies demonstrating that most ictal parameters are negatively associated with higher age [24, 30]. However, elderly patients often show a better response to ECT treatment [31]. This contradicts a monocausal assumption that ictal parameters could be non-age corrected predictors of clinical response [32]. This is underlined by results showing that the variance of antidepressive efficacy is only modestly explained by ictal EEG parameters [33].

SQI can be criticized for other aspects influencing seizure organisation and development. Stimulation dose, placement of electrodes (unilateral, bilateral), pulse width [26], concomitant drugs [34], time between injection of the hypnotic agent and stimulation [15–17], or hyperventilation [15, 35] may serve as examples.

Time in recovery room was primarily increased by higher age and not by S-ketamine dose. Age as a predictor of time staying in a post anaesthesia care unit is well accepted. Our finding regarding S-ketamine must not contradict a previous meta-analysis concluding that recovery time is prolonged (by only a few minutes) for patients receiving add-on ketamine compared to patients treated with other hypnotic agents [20]. This might be explained by the fact that we lowered both S-ketamine and propofol dose—thus dose ranges are different. Additionally, it might be explained by age, which was included as a covariate in our study.

Higher dose of S-ketamine (mg/kg) was positively correlated with the need of more urapidil (an alpha-1-antagonist) mirroring earlier findings [19].

The high number of ECTs per patient is explained by the inclusion of four patients (corresponding to 172 ECTs) receiving maintenance ECT and a significant proportion of patients with schizophrenia, who also received a higher average of ECTs per treatment period compared to patients with depression.

Our data are of retrospective character. Patients underwent multiple psychopharmacological therapy and changes within the ECT period. The patients’ diagnoses indicating ECT treatment (e.g. unipolar/bipolar depression, schizophrenia, catatonia), as well as pre-existing cognitive impairment, were not considered in our analysis and potentially biased our findings. Since this was a purely retrospective analysis, there was no protocol or standardized procedure on how the “clinical optimisation” process of the selection of dose and ratio of S-ketamine and propofol was exactly determined. Besides, we focused on seizure quality defined by ictal parameters without referring to its impact on clinical outcome, such as scaling of depressive symptom changes. Thus, it is not evident from this study how SQI and hypnotic agent’s doses might affect treatment response. Therefore, our results may not be generalizable to different clinical settings.

## Conclusions

To conclude, a S-ketamine: propofol ratio of 1.5 in favour of S-ketamine or a ketamine (racemate): propofol ratio of three in favour of ketamine has been empirically observed. Especially patients with poor seizure quality might benefit from the lower amount of propofol compared with a standard 1:1 mixture). Higher age turned out to correlate inversely with seizure quality and positively with time spent in the recovery room.
